# Poor resolution at the back of the tongue is the bottleneck for spatial pattern recognition

**DOI:** 10.1038/s41598-020-59102-3

**Published:** 2020-02-12

**Authors:** Zahide Pamir, M. Umut Canoluk, Jae-Hyun Jung, Eli Peli

**Affiliations:** The Schepens Eye Research Institute of Mass. Eye and Ear, Department of Ophthalmology, Harvard Medical School, Boston, MA USA

**Keywords:** Somatosensory system, Human behaviour, Vision disorders

## Abstract

Spatial patterns presented on the tongue using electro-tactile sensory substitution devices (SSDs) have been suggested to be recognized better by tracing the pattern with the tip of the tongue. We examined if the functional benefit of tracing is overcoming the poor sensitivity or low spatial resolution at the back of the tongue or alternatively compensating for limited information processing capacity by fixating on a segment of the spatial pattern at a time. Using a commercially available SSD, the BrainPort, we compared letter recognition performance in three presentation modes; tracing, static, and drawing. Stimulation intensity was either constant or increased from the tip to the back of the tongue to partially compensate for the decreasing sensitivity. Recognition was significantly better for tracing, compared to static and drawing conditions. Confusion analyses showed that letters were confused based on their characteristics presented near the tip in static and drawing conditions. The results suggest that recognition performance is limited by the poor spatial resolution at the back of the tongue, and tracing seems to be an effective strategy to overcome this. Compensating for limited information processing capacity or poor sensitivity by drawing or increasing intensity at the back, respectively, does not improve the performance.

## Introduction

Visual-to-tactile sensory substitution devices (SSDs) convey visual information to a blind person through touch. Since the seminal work of Bach-y-Rita in the 1960s^1^, tactile representation of visual information has been studied on various body parts including the back^1,2^, abdomen^3–5^, fingers^6–8^, and tongue^9–11^.

The tongue has been suggested to be a good platform for SSDs because it has higher sensitivity and spatial resolution compared to other body parts^9,12^. Users can recognize simple shapes, differentiate a small set of objects, detect motion, point to and avoid high-contrast-staged obstacles when the stimulus is presented sufficiently on the tongue (roughly 30 seconds)^9,13–17^. However, navigation and object recognition tasks in real-life situations, where the scene is usually dynamic, may require these tasks to be achieved at shorter times. Vincent, *et al*.^18^ showed that when a stimulus is presented for a short time (0.5 seconds), performance in discriminating basic shapes was not significantly above chance and performance in discriminating different line orientations deteriorated substantially compared to those reported by previous studies. They attributed the better performance in the earlier studies to the longer stimulation durations. This allowed participants to explore the stimulus actively and serially using the tip of the tongue (“tip” from here on), which may be required for spatial recognition through tactile input^19^.

Our experience with the BrainPort, an FDA-cleared electro-tactile tongue SSD (Wicab Inc., Madison, WI), is consistent with Vincent, *et al*.^18^. The BrainPort delivers the visual information acquired through a head-mounted camera to a 20 × 20 grid of electrodes on a 25.8 × 25.8 mm intra-oral device (IOD) placed on the tongue. Most blind and normally-sighted individuals we have trained on the device spontaneously (without explicit instructions) reported a strategy, where they traced the pattern on the IOD with the tip, especially in tasks like shape and letter recognition. Tracing with the tip was also recommended to users in two-point discrimination experiments^20^. However, to the best of our knowledge, the effect of tracing on recognition performance has not been systematically investigated before. We address two questions: (1) Does tracing with the tip of the tongue provide better performance over conditions in which tracing is not allowed, even if the presentation durations in the two conditions are matched? (2) What might be the underlying reason for users to intuitively implement an active/serial exploration strategy? Understanding the functional benefits of the tip tracing strategy might provide useful information about the tongue’s capability and limitations of processing spatial information, as well as guidance for device design and training.

The tongue is not uniform; the tip and its medial parts have higher sensitivity and spatial resolution than the back of the tongue (“back” from here on) and lateral parts, respectively^21–24^. If sensitivity or resolution on the tongue except the tip is insufficient to decode a spatial pattern presented on the IOD, then tracing with the tip might be an effective strategy to acquire all the information since it is the most sensitive region. This strategy resembles the use of saccadic eye movements in the visual system in bringing critical information to the fovea to compensate for the lower spatial resolution and contrast sensitivity at the periphery^25–27^.

Due to the brain’s limited information processing capacity^28^, even if a stimulus is large enough, relative to acuity, it is not always possible to comprehend all the information available at once. For instance, in visual recognition tasks, the number and duration of fixations increase as the complexity of a visual stimulus increases^29^. Similarly, tracing the pattern with the tip might be a fixation strategy. Recognizing complex spatial patterns on the skin is challenging due to the limited temporal and spatial resolution of the somatosensory system^19,30^. Dividing patterns into smaller parts might reduce complexity and focus the available resources to a smaller segment of the spatial input at any instant, thus improve recognition performance by serial processing but at a cost of increased time. Horner^31^ compared identification performance across simple (one-line) and more complex (two-line) spatial patterns presented vibro-tactilely on two fingers using Optacon arrays and showed that an increase in stimulus complexity impairs the identification performance (see also Horner^7^, and Craig and Evans^32^). Using a visual-to-auditory SSD, Brown and Proulx^33^ demonstrated that object recognition performance is more accurate and faster when the spatial information is divided into two parts and presented sequentially compared to when the entire pattern is presented simultaneously (see also Cronly-Dillon, *et al*.^34^ for using the same strategy).

Dividing the spatial pattern into smaller segments and displaying them sequentially on the finger, back or abdomen also improves recognition performance^2,3,35,36^. For instance, Loomis^2^ displayed uppercase letters on the back and compared four stimulation modes. In the first two conditions, the complete letter was presented at once either statically or moved across the back from right to left. In the other two conditions, he used a vertical slit/aperture to present only a part of a letter at a time. Either the slit was stationary and the letter was moving behind the aperture or the letter was stationary and the slit was moving in front of the letter. Recognition rates were significantly better in both of the slit conditions (51% and 47%) than in the conditions where the entire letter was presented at once (34% and 41%). Saida, *et al*.^3^ displayed Japanese syllabaries on the abdomen sequentially in a pattern that resembled hand lettering. This mode yielded superior recognition performance (95%) compared to when the entire letter was presented statically (20%) or moved horizontally (40%). Considering these findings, the functional benefit of tip tracing might be accomplished by dividing the pattern into smaller segments and fixating only one segment at a time to compensate for the complexity of the stimulus.

Using the BrainPort Vision Pro, we compared letter recognition among three conditions: tracing, static, and drawing. In the tracing condition, the complete letter was presented on the IOD and participants were instructed to trace the pattern with the tip. In the static condition, the complete letter was presented on the IOD, but participants were not allowed to move their tongue around the IOD (tracing with the tip of the tongue was not allowed). In the drawing condition, the letter was divided into smaller segments that were sequentially presented without removing the previously presented segments as in hand lettering and participants were not allowed to move their tongue around the IOD. This condition enabled fixation on a smaller segment at a time while presenting the stimulus on different parts of the tongue. Presentation duration for all conditions was set by the length of the drawing condition; it differed for each letter but was kept constant across the conditions for each letter.

We hypothesized that recognition performance will be better in the tracing condition than the static condition. If the functional benefit of tip tracing is dividing the pattern into smaller segments and the non-uniformity of the tongue does not have an effect, the performance in the drawing and tracing conditions should be similar and better than those in the static condition, as in the previous studies on other body parts^2,3,35,36^. If there is little or no difference in performance between static and drawing conditions while tracing yields a superior performance, the results will indicate that the performance might be limited by poorer sensitivity or spatial resolution on other parts of the tongue. Since some segments of a letter are presented on these parts with poorer sensitivity and resolution in the drawing condition, the recognition performance does not improve as a result of sequential presentation unlike the previous studies that used more uniform tactile surfaces^2,3,35^. In the experiment, we either kept the stimulation intensity constant or increased it (i.e., adjusted intensity) from the tip (lower intensity) to the back (higher intensity) based on the individual sensitivity of participants, to partially overcome the variability in sensitivity across the tongue. A change in results between the constant and adjusted intensity experiments will indicate the effect of non-uniformity in sensitivity.

We further predicted that if the recognition performance is limited by either sensitivity or spatial resolution, the patterns on the tongue can be effectively accessed only through the tip, which has the highest sensitivity and spatial resolution. If this is the case, letters are likely to be recognized and confused on the basis of characteristics at their lower part (presented on the tip) rather than the upper (presented on the back) in conditions where tracing with the tip is not allowed.

## Methods

### Participants

Sixteen participants were enrolled, eight of them were normally-sighted (6 women; mean age 27, *SD* = 2.9) and eight were blind (3 women; mean age 50.3, *SD* = 15.5). None of the participants had a history of any tactile impairment or oral pathology. Four participants were congenitally blind and had no residual vision, while four became blind after years of seeing, and two had light perception. Two normally-sighted and three blind participants were not available to participate in one of the two experiments. Eleven participants completed the whole study. Six were normally-sighted (4 women; mean age 26.5 years, *SD* = 2.6) and five were blind (2 women; mean age 56.8 years, *SD* = 16.5). All participants gave written informed consent. Experiments adhered to the tenets of the Declaration of Helsinki and the protocol and written consent were approved by the Massachusetts Eye and Ear Human Studies Committee.

### Apparatus and stimuli

The BrainPort Vision Pro translates images, either captured directly from a head-mounted camera or generated on a computer, into the electrical stimulation on the tongue (in the default configuration of the BrainPort Vision Pro, images are streamed only from the camera. However, the manufacturer provides another version of the device, for investigational use only, which enables direct streaming of image sequences from a computer using Wi-Fi connection. Otherwise, the investigational device is the same as the consumer device).

In conventional use of the BrainPort, a camera is mounted on the head and the head tremor caused by natural motion (i.e., jitter) is added to the images. It has been shown that jittering may improve low vision reading performance^37^. Therefore, sending computer generated images might not reflect the real performance with the head-mounted camera. To determine the effect of jittering on recognition performance, we conducted a pilot experiment to test the effect with simple lines and letters. Jittering did not significantly affect the performance (see Supplementary 1). Therefore, we used computer-generated static images streamed to the IOD for the present experiments.

We used letters as the spatial stimuli because they are complex in structure and less effort is needed to become familiar with the stimuli since most people, including most blind, are familiar with them. Uppercase English letters (26) were generated with white pixels on a black background in Sans Serif font using the open-source software GIMP (http://www.gimp.org/). The BrainPort Vision Pro’s IOD has 394 electrodes (20 by 20) and at the bottom corners of the IOD array, three electrodes are missing on either side. In order to avoid the missing electrodes, we did not use the first and the last columns or the bottom row of electrodes. In this configuration, the size of the letters was selected to fit up to 18 electrodes in a row. When the largest letter (W)’s size was adjusted to occupy 18 electrodes in a row, its height occupied 15 electrodes in a column. Heights of all letters were kept constant at 15 electrodes. Letters were centred laterally and aligned to the bottom (tip of the tongue). Therefore, no stimulation was presented on the top four rows of the electrodes (that stimulate the back). After creating an image file for each letter, we converted them into a 20 × 20 matrix of grayscale pixel values using MATLAB (R2017b). The matrix was then written to a text file, which was streamed to the BrainPort. Each cell in the text file stores the intensity value to be sent to one electrode.

The intensity of electro-tactile stimulation on the IOD depends on the stimulus intensity (0–255) and the intensity setting of the device (0% to 100%). Depending on each user’s tongue sensitivity, some intensity levels may be too weak for stimulus detection and others may be too strong to be tolerated. Therefore, before usage, stimulation intensity should be adjusted individually to a comfortable level. However, when the stimulation intensity is constant across the IOD, as in the conventional design of the device, a comfortable level of stimulation intensity for the back may be painful for the tip. Similarly, a comfortable intensity level for the tip may be weak or even undetectable for the back.

**Figure 1:**
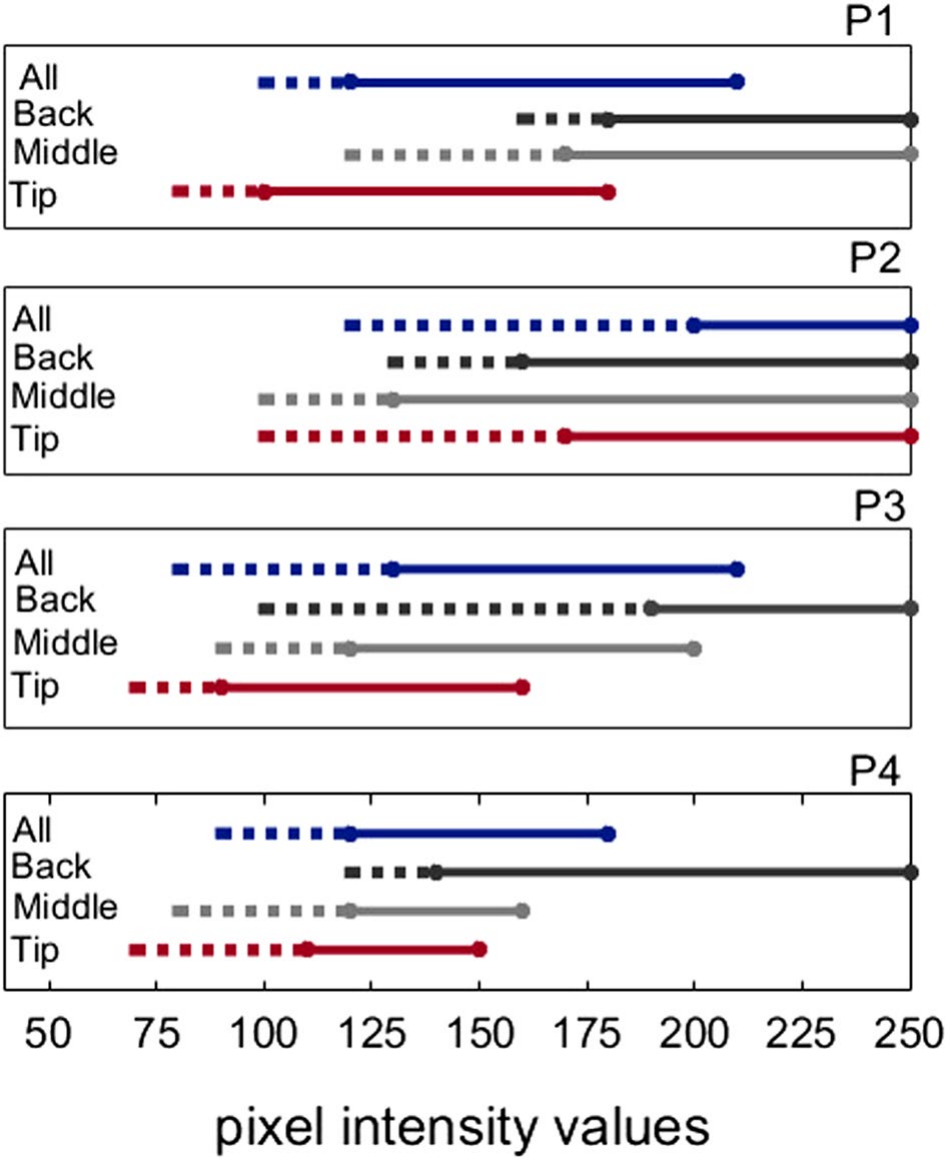
Stimulation intensity ranges for four participants (P1-P4) along different parts of the tongue. Either all the electrodes on the IOD were stimulated (All), or the stimulation was delivered locally to the tip, middle or back of the tongue. Stimulation intensities reported as either weak or comfortable were considered as the usable range. Dashed lines show the range that participants reported as weak (above detection threshold) and solid lines show range reported as comfortable, below pain level.

To determine the extent of the variation in sensitivity along the tongue, we conducted a pilot experiment with four participants (see Supplementary 2 for details). We examined the perceived stimulation intensity as a function of intensity values. First, all the electrodes on the IOD were stimulated with the same stimulation intensity within each trial. Participants reported the perceived stimulation intensity by selecting one of four pre-defined categories: no stimulus, weak, comfortable, and too strong (i.e. painful) sensation. In a separate session tip, middle, and back of the tongue were stimulated locally in separate trials with a constant intensity using a horizontal bar stimulus (20 × 6 electrodes). The usable range varies considerably across participants and different parts of the tongue. For all four participants, sensitivity decreases from tip to the back of the tongue. Also, when all electrodes were stimulated, the comfortable stimulation level range shifted slightly towards higher levels compared to stimulation at the tip of the tongue. For all participants, the lowest stimulation intensity level detected at the back was within the weak or comfortable range for the tip, middle, and all-electrodes stimulations (Fig. 1).

**Figure 2:**
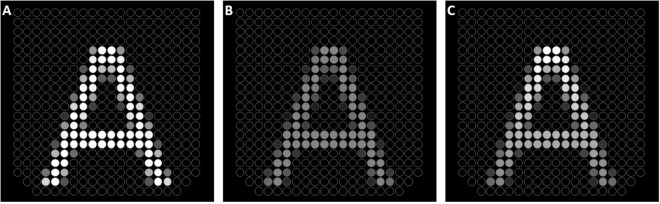
Stimuli with constant and adjusted intensity. Each circle represents an electrode on the IOD. The electrical stimulation intensity is illustrated by the pixel grey value assigned to each circle (when hardware intensity is adjusted to 100%, black and white correspond to no-stimulation and the maximum intensity, respectively). (**A**) Stimulus with a constant full intensity from the tip (lower part of the letter) to the back of the tongue (upper part of the letter). Some pixels were assigned grey levels for anti-aliasing. Stimulation intensity by this illustrated stimulus has been reported as painful by all participants. (**B**) Constant intensity stimulus with intensity adjusted to a comfortable level for each participant. (**C**) Adjusted stimulation intensity from tip to the back of the tongue. Less sensitive back of the tongue is stimulated with higher intensities. The higher intensity at the back of the tongue was not painful.

When users are asked to adjust the stimulation intensity to a comfortable level they likely make their judgements based on the sensation at the most sensitive part, the tip of the tongue. The results of our pilot sensitivity experiment showed that the comfortable stimulation level range of the tip overlaps at least with weak stimulation on the back of the tongue where the sensitivity is lowest. This means that even if users adjust the comfortable intensity based on the sensitivity on the tip of the tongue, stimulation intensity will be above the detection threshold for the back of the tongue. However, the effect of the difference between perceived intensity levels on the tip and back of the tongue on recognition performance is unknown. To examine if different perceived intensity levels on the tip and the back affect performance, we conducted two experiments; one with constant intensity and one with adjusted intensity (Fig. 2). In the constant intensity experiment, stimulation intensity was the same across the IOD. Before the experiment, we presented two vertical white bars (255 pixel intensity values; each bar’s size is 5 × 20) with a 5-pixel distance between them on the IOD and asked participants to adjust stimulation intensity of the device to a comfortable level. Participants were allowed to move their tongue on the IOD during the intensity adjustment.

In the adjusted intensity experiment, to partially compensate for the different perceived intensity levels along the tongue we increased the stimulation intensity from tip to the back of the tongue based on the preference of individual participants (Fig. 2C). We asked participants to adjust to comfortable stimulation intensity for the tip of the tongue and back of the tongue, separately. We presented a horizontal white bar (with pixel intensity of 255, 18 × 2 electrodes) at the back and at the tip in separate trials and asked participants to adjust the hardware intensity to a comfortable stimulation level. As mentioned above, stimulation intensity is proportional to the multiplication of stimulus intensity (pixel values) and hardware intensity. A white bar (with pixel value of 255) with 50% hardware intensity yields the same stimulation intensity with a stimulus whose pixel value is 128 when the hardware intensity is set to 100%. Since the system does not allow different hardware intensity values at individual electrodes, we set the hardware intensity to 100% and set the stimulus intensity for the bottom and top row of stimulus depending on the measured comfortable hardware intensity levels. Intensity value of each row between the bottom and top rows were set linearly along the vertical dimension between the comfortable levels for the tip and the back.

### Experimental design and procedure

**Figure 3:**
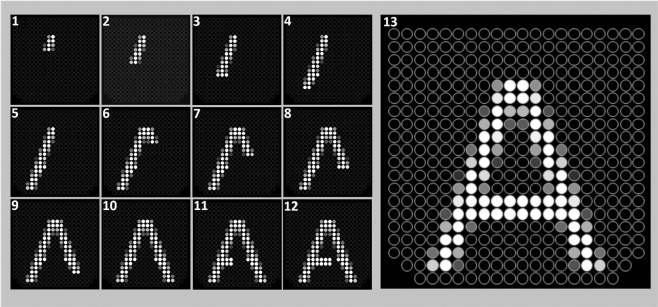
Letter ‘A’ stimulus used in the experiment with constant intensity. Each of the 13 frames represents segments stimulated on the IOD for 0.5 s in the drawing condition. Numbers show the presentation order. The last frame of the drawing condition (13) also shows the complete letter presented in the tracing and static conditions. The lower and upper parts of a letter are presented on the tip and the back of the tongue, respectively.

In the tracing and static conditions, the whole letter was displayed on the IOD simultaneously. In the drawing condition, each letter was divided into small segments that were presented sequentially. Drawing of an entire letter stroke was completed before starting the next stroke. Also, strokes that share common characteristics (e.g., horizontal or vertical strokes) were drawn sequentially. For example, the drawing sequence for the letter “A” is shown in Fig. 3. The drawing of letters such as “C” or “G” started from the top right end and was drawn counter clockwise towards the bottom. If there was no defined top endpoint as in the case of “O” or Q”, drawing started from the middle of the upper part.

The size of each segment of the letters was selected arbitrarily (i.e., 2 × 3, 3 × 3 or 4 × 3, the minimum and maximum number of pixels in a row or column was set to be 2 and 4, respectively) not to lose any pixel from the original font. Each segment was presented for 0.5 seconds (s), and since each letter has a different number of segments, completing each of them required a different duration. The shortest duration (3.5 s) was for the letter “J” divided into seven segments, while the longest duration (9 s) was for the letter “B” divided into 18 segments. The mean letter drawing duration was 6.3 s. In each trial, the letters were drawn only once in the drawing condition. The presentation duration set by the drawing condition for each letter was used in the other two conditions tested.

Before the session, normally sighted participants were blindfolded. All conditions and letters were counterbalanced and tested in a single session that lasted approximately two hours. There were 78 trials in total (26 letters × 3 conditions). In each trial, the letter and the condition were selected randomly. Before each trial, the participants were informed about what the next condition will be using an audio file which says “static”, “drawing”, or “tracing”. After the auditory cue, they started the stimulation by pressing a button on a computer gamepad. Participants were allowed to repeat each trial (letter presentation) up to three times if they were not confident about the correct response. There was a minimum of five seconds interval with no stimulation between each repeat and trial. After the interval, participants either repeated the same trial or moved on to the next trial when they were ready. Participants named the letter verbally and the response was recorded by the experimenter.

In the adjusted intensity experiment, the experimental design and procedure were the same as the constant intensity experiment except that we did not test the tracing condition. Since only the tip of the tongue is used to explore the stimuli in the tracing condition, there is no need for intensity adjustment.

### Training

Before the experimental sessions, all participants were trained on the device in various tasks such as spatial mapping, static bar orientation, localization, target counting, identification of shapes, letters and numbers, and recognizing 3D objects in space. The first four levels of the training protocol described in Nau, *et al*.^14^ were performed using the device’s camera. In addition to the general device training, they also completed a practice session designed to familiarize themselves with the experimental conditions of the present study.

In total, we have trained 29 blind individuals; and 17 of them could complete all steps in the training protocol. Other participants showed no progress even after 12 hours of training with the device or were dropped out of the study for reasons such as disinterest, inability to detect the highest stimulation level or keep the device in the mouth. Only eight of the blind participants who completed the training protocol (trained for minimum 6, maximum 33 hours) took part in this study.

Four normally-sighted participants were previously trained during the pilot testing of the device and completed all steps in the training protocol. The other four normally-sighted participants, who had no prior experience with the BrainPort, were first given basic training for an hour, which included the spatial mapping of the tongue in relation to the visual world (bottom-field: tip of the tongue, top-field: back of the tongue, etc.), and were presented bars both with the camera and with direct streaming in different orientations.

In the practice session, the conditions, experimental design, and procedure were the same as the experimental session, but 10 numerals instead of letters were used to avoid the familiarization effect with the actual stimuli. The stimuli were created in the same way as the letter stimuli. There were 30 trials (10 numerals × 3 conditions) presented randomly in each training block. Each participant completed a different number of trials in the practice session until they felt confident in their ability to perform in the experiment. Two out of 16 participants underwent more than one practice session. For the drawing condition, each segment in the practice sessions was presented for 0.35 s. BrainPort Vision Pro streams computer-generated images as frames and the presentation duration of each frame is 0.05 s (BrainPort Vision Pro user manual states that the device is able to stream computer-generated images at 20 frames per second). Although the device does not provide us with any way of systematically track the timing of frames, the stimuli being presented on the IOD can be observed in real-time using the monitoring software provided by the company. During our practice sessions, we realized that some of the segments were skipped and were not shown on the software screen when we present each segment for seven consecutive frames (0.35 s in total). Although this may not necessarily mean that those frames were not streamed to IOD, we decided to present each segment for 0.5 s in the experimental sessions to avoid skipping of an entire segment.

### Data analysis

Percent correct, and mean number of trial repetitions in correct responses data for each condition were analysed by applying mixed-design analysis of variance (ANOVA) with one within-subject factor (presentation mode) which has 3 levels (static, drawing, and tracing) and one between-subjects factor, sightedness (blind and normally-sighted) using SPSS Version 19 (SPSS Inc., Chicago, IL). Planned pairwise comparisons included tip tracing vs. drawing; tracing vs. static, and drawing vs. static conditions.

Percent correct, and mean number of trial repetitions in correct responses were compared between the constant intensity and adjusted intensity experiments by applying a mixed-design ANOVA with two within-subject factors (presentation mode and stimulus intensity mode) each of which has two levels (static vs. drawing; and constant intensity vs. adjusted intensity, respectively) and one between-subjects factor, sightedness (blind and normally-sighted) using SPSS.

### Confusion matrix analyses

We hypothesized that if the recognition performance is limited by either sensitivity or spatial resolution, letters are likely to be recognized and confused on the basis of characteristics at their lower parts (presented on the tip), rather than the upper (presented on the back) in static and drawing conditions where tracing with the tip is not allowed. To investigate this further we grouped the letters based on the characteristics at the lower and upper parts of the letters and created confusion matrices for each group to compare confusions within and between the groups, separately for lower and upper parts’ similarities. To group the letters based on lower part similarity, we covered the top half of each letter and only looked at the lower part. When we examined the lower half, we observed that each letter either has a single segment from a single stroke i.e., “F, I, P, T” or a single segment from two strokes that combine in the middle such as “V and Y”, or two discrete segments on both sides such as “A, H, K, M, N, R, W, and X”, or a horizontal segment (either straight or curved) extending from left to right such as “B, C, D, E, J, G, L, O, Q, S, U, and Z”. Using these characteristics, we defined three groups of letters by lower part similarity: one-, two-, and horizontal-segment group. We hypothesized that if participants are not sure about the correct response but can determine that there is only one segment at the lower part of the letter based on the information presented on the tip of the tongue, they would choose one of the letters from the one-segment group as their answer instead of giving a completely random answer. Therefore, their incorrect responses would be mainly letters from the same group that the target letter is part of.

We applied the same criteria for similarity at the upper half of the letters. For the upper part similarity, the one-segment group consisted of the letters “A, I, J, and L”; two-segment group consisted of the letters “H, K, M, N, U, V, X, and Y”; and the horizontal-segment group consisted of letters “B, C, D, E, F G, O, P, Q, R, S, T, and Z”. We hypothesized that similarities between the characteristics at the upper parts of letters will not affect the incorrect responses as the information at the back is not completely accessible because of poorer resolution and sensitivity at the back of the tongue. A letter that has one-segment at the upper part will be as likely to be confused with either one-segment or two-segment letters.

For each participant, we computed the percentage of times that letters in a group were confused with letters in that same group (within-group confusion) and with letters in other groups (between-group confusion). Since each group has a different number of letters, we normalized the percentage values by dividing the values by the number of letters in each group. For example, for letters in the one-segment at the lower part group, we computed the percentage of times letters in this group were confused with other one-segment at the lower part group letters (within-group confusion) and normalized the value by dividing it by 6 (number of letters in one-segment bottom group), with two-segment group letters (between-group confusion) and normalized the value by dividing it by 8, and with horizontal-segment group letters (between-group confusion) normalized the value by dividing it by 12. We replicated the entire procedure for the upper part similarity groups.

For each participant, we averaged the three within-group confusion scores and the three between-group confusion scores across groups for the lower part and upper part similarity groups separately and then subtracted the average between-group confusion from the average within-group confusion. A value of “0” corresponds to no difference between within-group and between-group confusions. Positive (negative) values mean within-group confusion is higher (lower) than the between-group confusion. We predicted that if the spatial information is available mainly through the tip of the tongue, within-group confusion will be higher than between-group confusion for the lower part similarity group. Therefore, within-group vs. between-group differences would be significantly higher for the lower part group than the upper part group. Also, there would not be a significant difference between lower and upper part similarity groups for the tracing condition because both lower and upper parts of the letters are explored through the tip of the tongue. We compared the differences in within-group and between-group confusions for lower and upper similarity groups by applying paired-samples Student’s t-test using SPSS.

## Results

**Figure 4:**
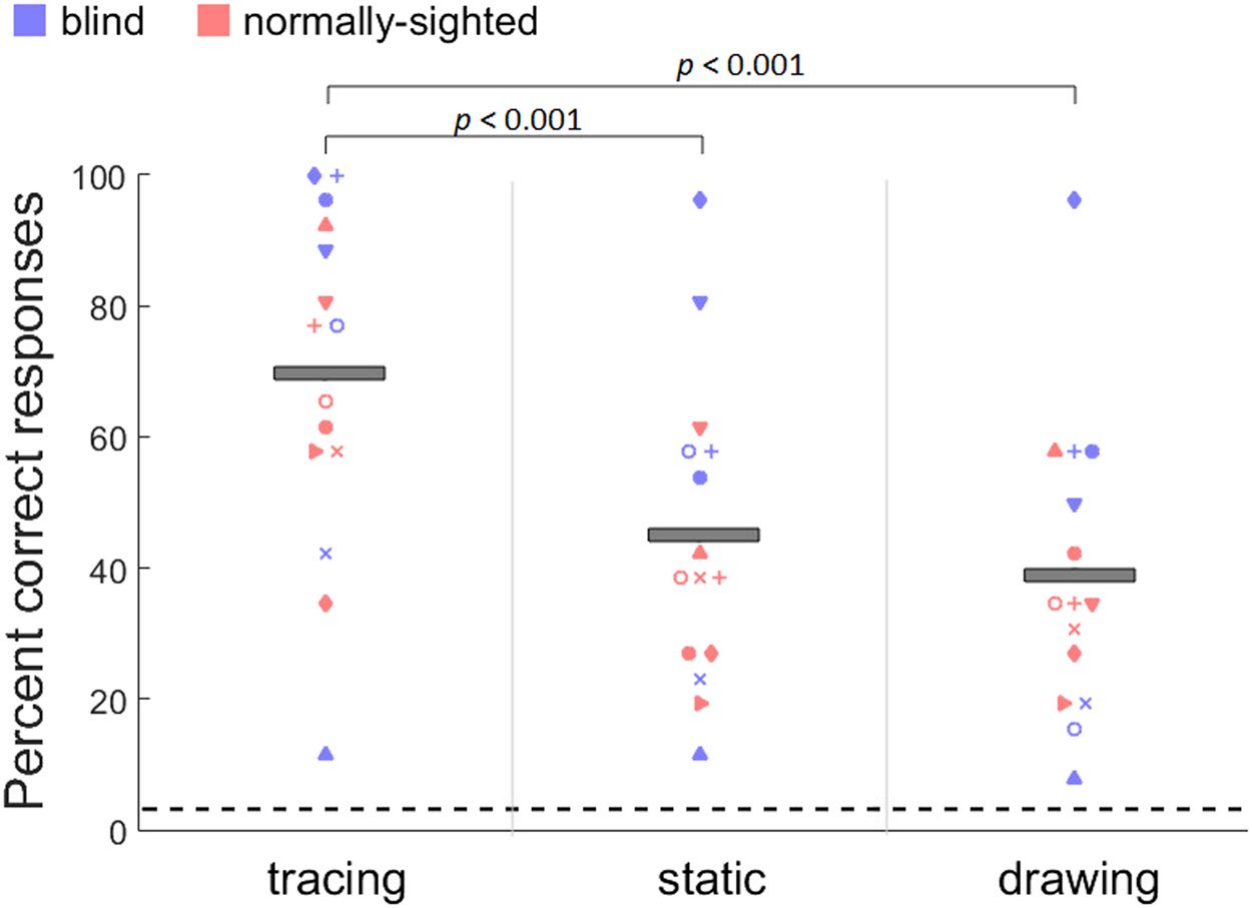
Individual and mean (grey bars) percent correct responses for the letter recognition in tracing, static, and drawing conditions. Performance is well above chance (horizontal dashed line: 1/26) for all conditions and participants. Tracing yielded significantly better performance than static and drawing conditions. There was no significant difference between static and drawing conditions. The trend in the data was similar for the blind and normally-sighted participants.

The individual and group mean percent correct responses collected from 15 participants for the constant intensity experiment are shown in Fig. 4. Results showed a significant main effect of presentation mode (*F*(2,28) = 30.4, *p* < 0.001). Bonferroni-corrected pairwise comparisons (corrected p-value is 0.016) showed a significant difference between tracing and static (two-tailed: *t*(14) = 6.04, *p* < 0.001), and tracing and drawing conditions (*t*(14) = 7.14, *p* < 0.001). Recognition performance was significantly better for the tracing (69%, SEM = 6.7) compared to both static (45%, SEM = 6.0) and drawing (39%, SEM = 5.8) conditions. There was no difference between the static and drawing conditions (*t*(14) = 1.44, *p* = 0.17). Also, the main effect of sightedness was not significant (*F*(1,13) = 0.98, *p* = 0.34) indicating that normally-sighted and blind participants did not perform differently.

**Figure 5:**
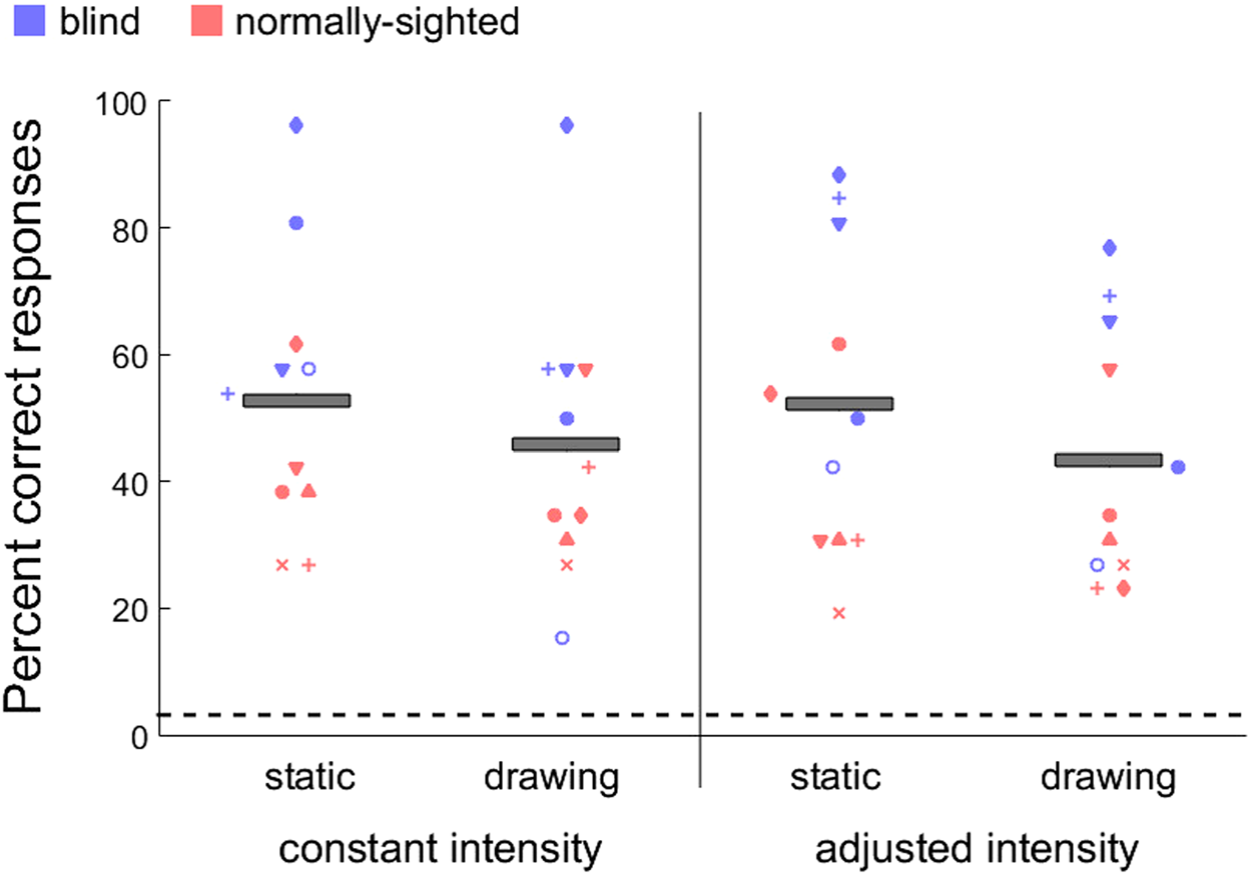
Individual and mean (grey bars) percent correct responses for the letter recognition in static, and drawing conditions across constant and adjusted intensity experiments. Performance is well above chance (horizontal dashed line: 1/26) for all conditions and participants. There was no difference in recognition performance between constant and adjusted intensity experiments. Increasing intensity at the back of the tongue where sensitivity is lower did not improve the recognition performance.

The performance of the 11 participants who completed both the constant and adjusted intensity experiments was also compared (Fig. 5). There was no significant difference in the recognition performance between the stimulation with constant intensity (49.3%, SEM = 4.6) and adjusted intensity (47.7%, SEM = 4.6) (*F*(1,10) = 0.17, *p* = 0.68). Also, there was no difference between the static (52.4%, SEM = 4.8) and the drawing (44.5%, SEM = 4.3) conditions (*F*(1,10) = 3.08, *p* = 0.11). Blind participants significantly performed better than normally-sighted participants (F(1,9) = 8.54, p = 0.01). However, some blind participants who performed worse in the first experiment did not want to participate in the second session. Therefore, this sample for the blind might be biased.

### Number of repetitions

During constant and adjusted intensity experiments, participants were allowed to repeat each trial (letter presentation) up to three times. In most of the trials, for both experiments, participants preferred to repeat the trial with the maximum number of repeats.

The number of repetition analyses in the constant intensity experiment showed a significant main effect of presentation mode (*F*(2,28) = 4.32, *p* = 0.02). However, Bonferroni-corrected pairwise comparisons (corrected *p*-value is 0.016) showed no significant difference between tracing (M = 2.59, SEM = 0.09) and static (M = 2.77, SEM = 0.07) (*t*(14) = 2.52, *p* = 0.02), tracing and drawing (M = 2.76, SEM = 0.08) (*t*(14) = 2.2, *p* = 0.04) and static and drawing conditions (*t*(14) = 0.22, *p* = 0.8).

Results were similar in the adjusted intensity experiment, there was no difference in the mean number of repetitions that participants required in trials with correct responses for static (M = 2.67, SEM = 0.11) and drawing (M = 2.72, SEM = 0.08) conditions (*t*(10) = −0.7, *p* = 0.49).

### Confusion matrix analyses

**Figure 6:**
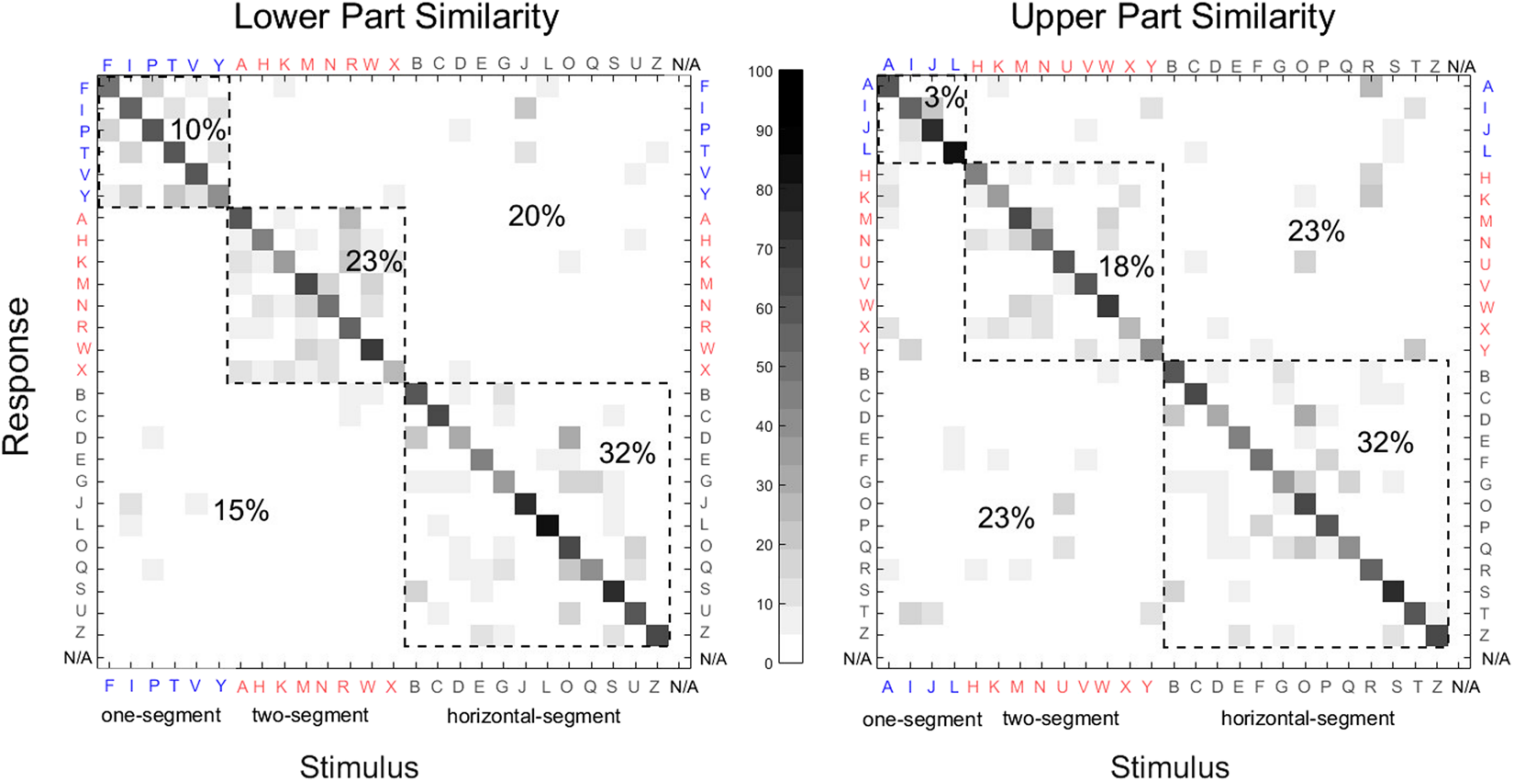
Confusion matrices for the letters grouped by stroke characteristics at their lower (left) and the upper (right) parts. The letters are grouped by characteristics marked and ordered in the figure accordingly as one-segment, two-segment, and horizontal-segment groups. Data within the dashed-line squares show the conditions where participants’ responses were either correct (along the diagonal), or incorrect, but the target letter was confused with a letter from the same group (within-group confusion). The areas outside of the dashed-line squares show the conditions where participants’ response was incorrect and the target letter was confused with a letter from the different groups (between-group confusion). The percent difference, normalized by the number of elements in each group, between the within-group confusion (65%) and between-group confusion (20% + 15% = 35%) was significantly higher (p < 0.01) for the letters grouped by the lower similarity (left) compared to those grouped by the upper similarity (right, 54% for within-group confusion, and (23% + 23%) 46% for between-group confusion). Note that the upper part of the letter is presented on the back of the tongue (lower resolution). The greyscale shows the percent response (both correct and incorrect). N/A shows the trials where participants did not want to give a response (3 trials in total and, therefore, not visible).

Confusion matrices created based on similarities at the lower and upper parts of letters are shown in Fig. 6. Since there was no difference between the static and drawing conditions, and constant and adjusted intensity conditions, we combined the data from 27 sessions by all 16 participants (11 participants participated in both experiments (22 sessions), four participated only in the constant intensity and one participated only in the adjusted intensity experiment) to create the confusion matrices and carried out analyses using the combined data. The difference between within-group confusion and between-group confusion was significantly higher for the lower similarity group than the upper similarity group (*t*(15) = 3.5, *p* = 0.003). Note that the upper part of a letter is presented on the back of the tongue (lowest sensitivity and resolution). Letters which have similar characteristics at the lower part were confused more often with each other (65% of all incorrect responses) compared to those that have different characteristics at the lower part (35% of all incorrect responses). In comparison, confusion rates at the upper part were similar for the letters which have similar (54% of all incorrect responses) or different (46% of all incorrect responses) characteristics. As predicted, there was no difference in within-group vs. between-group confusions between lower and upper similarity groups for tracing condition (this analysis includes data only from the constant intensity experiment; *t*(14) = 0.31, *p* = 0.76). These results further support the postulation that information available from the tip of the tongue significantly defines the participants’ responses.

## Discussion

Our results show that tracing the spatial pattern on an IOD with the tip of the tongue provides the best performance in recognizing letters. Unlike previous studies on the skin of the abdomen, back, and finger^2,3,35^, drawing the pattern on the tongue did not improve the performance compared to the static presentation with either constant or adjusted stimulus intensity. Since no difference was observed in performance between static and drawing conditions, the functional benefit of tip tracing does not seem to be purely derived from dividing the spatial pattern into smaller segments to compensate for the limited information processing capacity.

Why does drawing improve performance on other body regions but not on the tongue? One reason might be the variable sensitivity along the tongue. The back of the tongue has been reported to be approximately 30 percent less sensitive than the tip^22^. The result of our perceived intensity measurement indicates that stimulation intensity level comfortable/strong at the tip is above detection threshold at the back when these areas are stimulated separately. However, it is not clear how psychophysical properties such as adaptation or surround suppression affect the detection of stimulation presented on the back when there is stimulation elsewhere along the tongue. For instance, an interaction with a static stimulus triggers an initial sensation, but the magnitude of sensation drops almost immediately due to adaptation^38,39^. Since the perceived intensity is already low at the back, the sensation might fade below threshold just after the first interaction with the stimulus. In such a case, recognizing spatial details presented on the back may not be possible in static and drawing conditions. In order to rule out this possibility, in the adjusted intensity experiment, we increased the stimulation intensity from tip to the back based on the preferred intensity levels of each participant on the tip and back. Our results suggest that as long as the stimulation is above detection threshold, poor sensitivity does not significantly affect the performance on such binary stimuli. Therefore, the reason why drawing does not improve performance on the tongue cannot be attributed to poor sensitivity at the back.

Another reason why drawing improves performance on other body regions but not on the tongue might be related to poor spatial resolution at the back of the tongue. One support for this postulation comes from the previous findings that unlike the tongue, spatial resolution is relatively uniform on the torso^40^ on which sequential presentation of smaller segments improves performance. This finding points out that when spatial resolution is sufficiently high relative to stimulus across the body part surface where the stimulus is presented, sequential presentation improves performance. If poor resolution is the reason that impedes recognition in static and drawing conditions using the BrainPort, the resolution should be sufficient at the tip but poorer than required to recognize the stimuli used in our experiments at the back. Essick, *et al*.^41^ demonstrated that the average threshold size for recognizing raised letters (50% correct identification) is 5.1 mm (range 3.7 to 6.6 mm) when using the tip tracing. More recently, Miles, *et al*.^42^ measured the average threshold size as 4.54 (±1.41) mm on the tongue which is consistent with Essick, *et al*.’s^41^ results. The letter size that we used in the experiments was 19.2 mm, approximately four times larger than the average threshold reported in these studies^41,42^, thus the resolution of the tip should be sufficient to resolve the letters we presented in the experiments. However, if the difference in resolution between the tip and the back is larger than the factor of 4, resolution at the back will be insufficient for recognition of our letters without using the tip tracing. Previous studies reported that, on average, people can discriminate two distinct stimuli which are separated by 0.58–2 mm on the medial part of the tip (note that threshold is higher at the lateral parts)^12,43–45^. Although different studies agree that two-point discrimination sensitivity decreases from the tip to the back, they reported considerably different thresholds for the back of the tongue. For instance, Lass, *et al*.^44^ showed with mechanical stimulation that the mean two-point discrimination threshold at a medial point 30 mm posterior to the tip was 2.96 mm (range 1.05 to 6.75 mm), approximately 5–1.5 times poorer than the spatial resolution at the tip of the tongue. The size of each electrode on the BrainPort IOD is 0.76 mm in diameter, with a 0.56 mm separation between each. Lass, *et al*.’s^44^ results indicate that there should be at least two electrodes which are not stimulated (corresponds to 3.2 mm) between the two active electrodes in order to discriminate them successfully at the back. Moritz *et al*.^24^ reported that participants were unable to discriminate two discrete electrical stimulations in the region 20 mm to 40 mm posterior to the tip even with the largest distance tested in the experiment (7 mm from edge-to-edge of two active electrodes, at least 15–3.5 times larger than the tip’s resolution). Since Moritz *et al*.’s^24^ results do not provide a threshold for the back, we cannot infer what size gap would be sufficient to discriminate two distinct stimulations. However, we can at least predict that participants will not be able to discriminate two stimulations if there are fewer than five electrodes unstimulated (corresponds to 7.16 mm) between two active electrodes especially for stimulation from the top four rows of electrodes which we did not use in the experiment (stimulates the area beyond 20 mm posterior to tongue tip). Still, our results show that this calculation is valid for the remaining electrodes that we used to present letters on the back. For example, the two strokes of “H” were separated by seven electrodes in the present experiments yet it is most commonly confused with “R” likely because the two vertical strokes at the top are perceived as joined. Based on these calculations, the spatial resolution at the back appears to be poorer than required to resolve the stimuli we presented.

Letters are recognized based on global shape information and fine details^2,34^. If recognition is limited by the poor spatial resolution at the back, the main accessible information in the static and drawing conditions where tracing is not allowed is likely to be the global shape and the portion of the fine details presented on the tip. This might explain why tracing provides a superior performance over static and drawing conditions as the amount of accessible fine details is higher with tracing than in the other two conditions. If this is the case, in addition to the global shape information, letters are likely to be recognized on the basis of their characteristics at the lower part (presented on the tip) rather than at the upper part (presented on the back) in the static and drawing conditions. Our confusion matrices support this hypothesis by showing that letters are more likely to be confused with other letters which have similar characteristics at the lower parts (higher resolution) rather than those that have similar characteristics at the upper parts. It should also be noted that most of the letters are symmetrical at the lower and the upper parts (16 out of 26 such as ‘B, C, and E’). This may cause overestimation of the within-group confusions in the upper similarity groups. For instance, “D” might be confused with “O” just based on the similar information at the lower part presented on the tip, but because they are also similar on the upper parts, our confusion matrix analysis considers that this confusion is affected by the upper similarities as well. Therefore, the real effect might be larger than revealed by our analysis. Overall, our results indicate that tracing may be an effective strategy to overcome the poor spatial resolution at the back of the tongue by decoding the whole pattern (lower and upper parts) using the tip. In other words, spatial recognition is already limited by poor resolution at the back, therefore compensating for limited information processing capacity by presenting a smaller segment of a stimulus sequentially at a time does not improve performance.

Yet, another possibility is that bottom characteristics of letters might already be more informative than the characteristics at the upper part so that there is not much need to recognize the upper parts. Previous findings show that the upper parts of the mixed-case English letters are more informative than the lower parts^46^. However, the upper and lower parts of uppercase letters were equally informative^47^. Since we used uppercase letters in the experiment, it is unlikely that the present confusions are affected purely by the inherited characteristics of the letters.

It might be considered that active tracing with the tip provides better performance over drawing condition because of the kinaesthetic information supplied through the self-controlled motor movement. However, the results in the literature about the superiority of active over passive touch are inconsistent. Heller^48^ compared active touch with passive and passive sequential touch on the finger in a shape recognition task. These conditions were similar to our tracing, static, and drawing conditions, respectively. He found that active touch was significantly superior to both of the passive conditions and there was no difference in recognition performance between the two passive touch conditions, similar to our results. On the other hand, Vega-Bermudez, *et al*.^49^ controlled for the size of the stimulus and found that there was no difference between active touch and passive sequential touch conditions in a letter recognition task if letters were smaller than a finger pad. Similarly, Kudoh^35^ indicated that previous studies used relatively larger patterns that make using kinaesthetic information necessary and therefore possibly helpful. Kudoh^35^ tested the active, passive and passive sequential touch (referred to as passive scanning in the paper) modes using smaller letters presented on a single finger and found that active touch and passive sequential touch conditions yielded similar and superior performance than the passive touch condition. These studies by Vega-Bermudez, *et al*.^49^ and Kudoh^35^ indicate that kinaesthetic information acquired through self-controlled motor movement does not necessarily improve performance over a condition where there is no motor-control when the stimulus is smaller than the effective biological sensor on which stimulus is presented. The size of the letters we used in the experiment was smaller than the size of the IOD, which allowed the complete letter to be presented on the tongue at one time. If the whole region of the tongue on which the stimulus is presented was useful to decode the pattern, we would not expect to see a difference in performance between tracing and drawing conditions. However, since only the tip of the tongue seems to be the effective biological sensor which is sufficient to decode the patterns, tracing might be necessary to access all information provided by the stimulus.

Magee and Kennedy^50^ pointed out that active tracing might even decrease the performance presumably by increasing cognitive load as a result of additional requirements of the active condition such as planning and controlling for the movement. They compared the effect of active and passive tracing on recognition. In the active condition, participants were allowed to explore the stimulus freely by index finger whereas in the passive condition, the experimenter controlled the movement of participants’ finger. They found that passive tracing yielded better recognition performance than active tracing. They attributed the results to the increase in the task’s demands in active condition whereas in the passive condition, participants could focus only on recognition. Van Doorn, *et al*.^51^ replicated Magee and Kennedy^50^ experiment. Using fMRI, they measured blood oxygenation level-dependent (BOLD) activity during active and passive touch conditions. Active touch yielded higher BOLD activity than the passive condition in areas associated with monitoring and controlling of goal-directed behaviour, attention, execution of movements, and error detection. On the other hand, activity was higher for passive touch in the areas linked with touch perception, length discrimination, and tactile object recognition. They explained the superiority of passive touch by the negative effect of increased cognitive load in the active touch condition. In our experiments, cognitive load in the tracing condition is higher than the drawing condition because of the need for planning and movement control required during tracing. Therefore, we might expect to see better recognition performance for our drawing condition. However, we found the opposite effect. Considering that there is no difference in performance between active scanning and passive sequential presentation modes when the size of the stimuli is smaller than the electrode array, and cognitive load caused by active tracing negatively affects recognition performance, the superiority of the tracing condition in the present study is unlikely to be purely caused by the kinaesthetic information acquired through active scanning in the tracing condition.

One limitation of our study might seem to be the streaming method we used. Since we sent the images directly to the IOD, participants could not benefit from some advantages like super resolution due to jittering. However, the results of our pilot experiment showed that jittering did not affect the performance (see Supplementary 1). Besides, our main aim in the present study was to compare the presentation conditions. Even if there is an effect of direct streaming on the performance, all the conditions should have been affected by the direct streaming to the same extent. Therefore, we believe that the pattern of our results is not crucially affected by the streaming. Also, as mentioned in the methods section, we were limited by technical specifications of the device, especially in the drawing condition that we could not reduce the presentation time. However, we do not expect that shorter presentation times would improve recognition performance because Craig^6^ showed that increase in presentation duration of each segment improves performance (up to 0.4 s, there was little change in performance with further increases in presentation duration and we used 0.5 s).

One might argue that instead of presenting each segment in a cumulative manner as we did (see Fig. 3); presenting only one group of pixels at a time (deleting the previous one) simulates the tip tracing condition better in the drawing condition. However, since salient and relevant objects to the task capture attention in visual attention tasks^52^, we assume that attention should be captured by the newly presented segment each time when there is a change in the stimulation. Also, considering that tactile working memory is more limited than visual working memory^53^, presenting only one segment of a spatial pattern at a time might increase the task’s demands because participants would have to keep the position of previous segments in (spatial) memory. However, this is not exactly the case in the tracing condition where they can always go back to parts they previously explored as long as presentation time permits. Therefore, to decrease the working memory demand of the task and to give participants a reference we kept the previously presented groups of segments on the screen.

Although correct recognition performance in our experiment is well above the chance level for all conditions, it is less than 50% for drawing and static conditions and only around 70% for the tracing condition. Despite the unrealistic ideal experimental conditions of high contrast and clear background without any clutter, considering the time required to correctly recognize only a single letter 50% of the time (approximately 18 seconds), using tongue stimulation does not seem practical for tasks requiring complex spatial pattern recognition. Besides, in our experiments, participants were already familiar with the finite set of the stimuli that they will encounter. Therefore, top-down mechanisms are highly involved in information processing during the experiments, which might enhance perception by facilitating the prediction of the stimulus, as supported by the confusion matrix analyses. In daily usage, users should be able to recognize objects through mere bottom-up processing. Yet, to the best of our knowledge, there are no studies demonstrating bottom-up object recognition using tactile SSD which we would predict to yield worse recognition performance than those reported in the literature so far.

It has been argued that one of the main advantages of using tongue over fingers is the larger surface of the tongue^9,10,21,54^. However, our results suggest that poor resolution at the back of the tongue limits the efficient use of considerable fraction of the tongue’s surface and constrains the user to trace the pattern with the tip in order to reach to a performance level which is still far from practical. Using only the tip of the tongue as a platform for SSD might still be better than fingers because it has higher resolution than fingers and its size is comparable to a finger’s size, at least horizontally. However, if tracing is required for a reasonable performance, then tracing with a finger would be easier and faster because finger is more mobile compared to the tongue. Overall, the larger surface of tongue does not seem to provide a useful larger platform because of the non-uniformity in spatial resolution along the tongue (see also Vincent, *et al*.^18^).

## Supplementary information

Supplementary Material

## Data Availability

The datasets generated during the current study is available at https://pelilab.partners.org/.
