# Supplementary material for: Poor resolution at the back of the tongue is the bottleneck for spatial pattern recognition
Source: Sci Rep. 2020 Feb 12;10:2435. doi: 10.1038/s41598-020-59102-3 (PMC7015888; doi:10.1038/s41598-020-59102-3)
Supplement: Supplementary file 1 — Supplementary Material [file 41598_2020_59102_MOESM1_ESM.pdf]

Supplementary Material

Poor resolution at the back of the tongue is the bottleneck for spatial pattern recognition

Zahide Pamir, M. Umut Canoluk, Jae-Hyun Jung, Eli Peli

The Schepens Eye Research Institute of Mass. Eye and Ear, Department of Ophthalmology, Harvard Medical School, Boston, MA

1. Measurement of usable range of stimulation on the dorsal surface of the tongue

The intensity of stimulation on the intro-oral device (IOD) depends on pixel value of the image, and the intensity setting of the device (intensity knob) which can range from 0% to 100%. Stimulation is voltage controlled and is limited to 16.7 Volts (100% hardware intensity for stimulus with pixel value of 255). Each user adjusts the hardware intensity to a comfortable level (i.e., usable range, a range between detection threshold to painful level).

Considering the area covered by the electrodes ($26\times$ 26 mm; IOD size is 29.5 mm H $\times$34 V mm), sending a uniform signal to all electrodes on IOD, stimulates almost the whole anterior tongue part (anterior tongue is approximately 25 mm from tip towards back[1]). However, sensitivity is not uniform across the tongue[1,2]. The tip of the tongue is more sensitive than other parts. Therefore, a pixel value that can be detected by tip of tongue may not be detected when back of the tongue gets stimulated by the same intensity. In order to understand the relationship between stimulus strength and sensitivity of different parts of tongue, we investigated how the usable range changes when all electrodes or only a few electrodes located on tip, middle, or back of the tongue are stimulated.

Stimuli were 20×20 electrodes computer-generated images. Pixel values change from 50 to 250 in increments of ten pixel value steps (21 pixel values in total) tested in the experiment. Two experiments were conducted. In the first experiment, all 400 electrodes on the IOD were stimulated. In a second experiment, only upper, middle or lower parts of IOD (20×6 electrodes) were stimulated. We streamed the stimuli from the computer directly to the IOD in a randomized order and presented on the IOD for 3 seconds with the hardware intensity level set at 100%. There was a minimum of three-second inter-trial interval (ITI); next trial began whenever the participant was ready after the ITI. Each stimulus was repeated three times (63, (21×3) trials in the first experiment; and 189 trials in the second experiment).

Participants were asked to put the IOD flat on their tongue as it is the conventional use of the BrainPort. They were required to rate the stimulation intensity by selecting one of the four pre-defined categories (i.e., not detectable, weak, comfortable, too strong). The category that participants report at least twice out of three times for a particular stimulation level is recorded as the category that stimulation level belongs to. If there was no consistent answer at least two times; we recorded that pixel value as in the same category with the neighboring pixel value one step below. The results are present in Fig. 1 in the paper.

2. Testing the effect of jitter on recognition performance

BrainPort Vision Pro enables to stream visual information gathered through an embedded live camera or as computer-generated images without using the camera. Performance across two streaming conditions may not be comparable. For instance, while the visual information is gathered through a live camera, users wear the device on their head and even if they aim at a particular object the stimulation on the tongue moves slightly due to the inevitable small head tremor. While using a static computer-generated image causes it to always be represented in the same location on the tongue regardless of head tremor. Because head tremor (jitter) slightly changes the location stimulated (moving target across the head movement), it might improve the performance by preventing the stimulation from fading caused by the fast adaptation properties of the receptors on tongue [3,4].

It previously has been shown that jittering helps to better recognize spatial patterns presented on fingertips[5]. Also, it has been shown to enhance detecting changes in the spatial pattern only after a long duration (~10 secs) of electro-tactile stimulus presentation on the tongue [6]. However, we do not have any clear evidence how jittering affects recognition of electro-tactile stimulation on the tongue when there is no change in the spatial pattern across time. In order to elucidate if jittering caused by the small head movements during the conventional use of BrainPort helps to recognize stimuli faster or with a better accuracy, we simulated jittering in the direct streaming condition and compared performance across static and jitter conditions.

a. Testing the effect of jitter on recognition performance with bars

Methods

Participants

Nine participants took part in the experiment (including authors ZP and MUC). Participants' mean age was 30.8 (*SD* 8.60). No participants had history of any tactile impairment. One participant was blind. Normally sighted participants had no history of visual impairments other than the corrected refraction errors. All participants gave written informed consent. Experiments accorded with the Declaration of Helsinki and the protocol and written consent were approved by the Massachusetts Eye and Ear Human Studies Committee.

Apparatus & Stimuli

Stimuli were bars in four different orientations; horizontal, vertical, left- and right-tilted (Fig. S1). Bars were located on different parts of the IOD in order to prevent participants to make educated guesses based only on either the starting or ending point of the stimulus without actually recognizing the orientation. Also, in order to see the effect of tilt level, both 22.5 and 45 degree tilted bars from vertical direction were used in the experiment. 22.5 degree tilted bars were shown in two different locations; top part is either at the center or closer to the edges of IOD (e.g., if it is left-tilted its top is closer to the left edge of the IOD). Because of the limited spatial resolution, 45 deg. tilted bars could only be shown in one location.

Two conditions were tested in the experiment; static and jitter. In the static condition, the stimulus was statically presented on IOD throughout the trial. The first and last two electrodes of each row and column on IOD were always left empty in the static condition, therefore the static stimulus only occupied 16x16 electrode array to clear adequate space in the jitter condition (e.g.; a horizontal line

started from 3rd pixel from the left and ended at 18th pixel). In the jitter condition (Fig. S2), the aim was to simulate the natural jitter that is caused by the movements of the head when visual input comes through the camera. The position of each stimulus in the static condition was considered as the baseline position and the electrode at the center of the baseline position and the eight electrodes surrounding it were defined as the jittering locations. The center of the stimulus was shifted to one of these nine pre-defined locations in every frame in a randomized order. Note that the stimulus does not have to start from the baseline position or to go back to the baseline position after each movement.

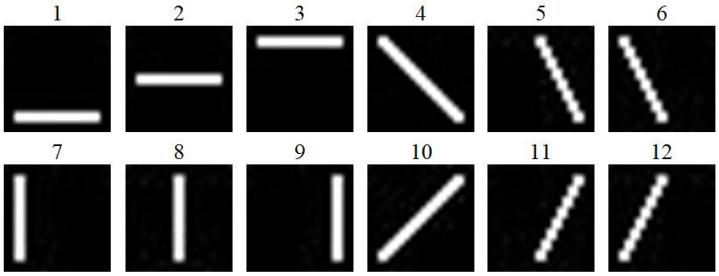

Figure S1. Stimuli used in the experiment. **1-3:** Horizontal lines in 3 different locations. **4:** Diagonal line with 45 deg. tilted to the left. **5-6:** Diagonal lines with 22.5 deg. tilted to the left, in 2 different locations (top is closer to the center and to the edge, respectively). **7-9:** Vertical lines in 3 different locations. **10:** Diagonal line with 45 deg. tilted to the right. **11-12:** Diagonal lines with 22.5 deg. tilted to the left, in 2 different locations.

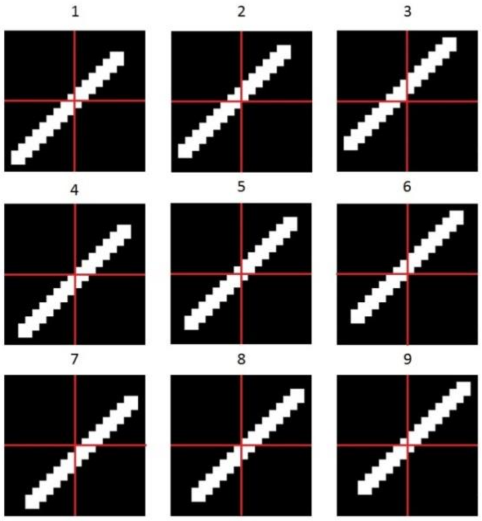

Figure S2. Nine pre-defined locations where a diagonal bar is presented in the jitter condition. The position of each stimulus in the static condition was considered as the baseline position (5), and the electrode at the center of the baseline position and the eight electrodes surrounding it were defined as the jittering locations. Note that the red cross on each image is not present in the original stimulus. The stimulus' relation to the red cross' center can be used as a cue to understanding the changes in a location better. The center of the stimulus was shifted to one of the nine locations every ~50 milliseconds in a randomized order. Note that the stimulus does not have to start from the baseline position or go back to the baseline position after each movement.

Procedure & Data Analysis

One blind and five normally sighted participants had prior experience with the BrainPort device. The blind participant previously had 10-hour training, in three different sessions, to participate in another BrainPort study. Five normally sighted participants were previously trained during the pilot testing of the device. The remaining three normally sighted participants, who had no prior experience with the BrainPort, had taken the basic training of at least 1 hour, which included the space mapping of the tongue in relation with the visual world (bottom-field: tip of the tongue, top-field: back of the tongue, etc.), and were shown basic bars in different orientations to make sense of how they are represented on tongue. The training session was immediately followed by the experimental session. Before starting the experiment, the stimuli were introduced visually to the normally sighted participants and verbally to the blind participant, and the task was explained. Afterward, normally sighted participants were blindfolded, and all participants were asked to put the IOD on the dorsal surface of their tongue and instructed not to move their tongue to use only some parts of it (such as using only tip of the tongue) to explore the stimulus. An example stimulus is then presented on IOD to help the participants with adjusting electrical stimulation intensity to a robust but not an uncomfortable level.

In the experimental session, participants were required to start and stop the stimulation for each trial by pressing a button on a computer gamepad. Before each trial, a beep sound was given to indicate that they can start the trial whenever they are ready. The time interval between the start and stop presses were recorded as response time (RT). They gave responses after they stopped the stimulation either verbally or by drawing/showing the orientation with their arm/hand, and the response was recorded by the experimenter. Each condition was repeated five times. There were 120 trials in the experiment (4 orientations × 3 positions × 2 motion conditions (static vs. jitter) × 5 repetitions). The data were collected in a single experimental session that lasted approximately 30 minutes.

Accuracy, and mean RT of correct responses data were analyzed by applying repeated measures analysis of variance (ANOVA) with two factors (orientation and motion condition; static vs. jitter). Further, static and jitter conditions were compared using two-tailed paired-samples Student's t-tests. Jamovi open source data analysis software (www.jamovi.org/) was used to analyze the data. Also, the effect of position for each orientation condition was analyzed using one-way ANOVA.

Results

Mean percent correct responses data is shown in Figure S3. There was no statistically significant difference between static and jitter conditions ($F(1,8) = 0.17$, $p = 0.69$). However, there was a main effect of orientation ($F(3,24) = 7.4$, $p = 0.001$). Horizontal and vertical bars were recognized better than the left and right tilted bars. Performance was the worst when 22.5 deg. left tilted bars's top is close to the edge. There was no interaction between motion condition and orientation ($F(3,24) = 1.31$, $p = 0.29$). When we compare each condition for the effect of motion, the only significant difference was between static and jitter condition was seen when vertical bars were presented on the right side of the tongue ($t(8) = 2.5$, $p = 0.03$, not significant after Bonferroni correction). In fact, jitter yielded a lower performance in this condition. Because there was no difference between two motion conditons in general, we averaged the results of static and jitter conditions and compared the effect of position for the further analysis. Results showed that there is no main effect of position either for horizontal ($F(2,16) = 0.15$, $p = 0.86$) or for vertical bars ($F(2,16) = 0.6$, $p = 0.56$). However, for the left- and right-tilted bars, position affected the performance ($F(2,16) = 15.6$, $p < 0.001$; $F(2,16) = 7.73$, $p = 0.004$, respectively). Both for the left- and right-tilted bars, the highest

performance was observed for the 45 deg. tilted bars and the lowest was for the 22.5 deg. tilted bars whose top is closer to edges, i.e. away from the center of the tongue.

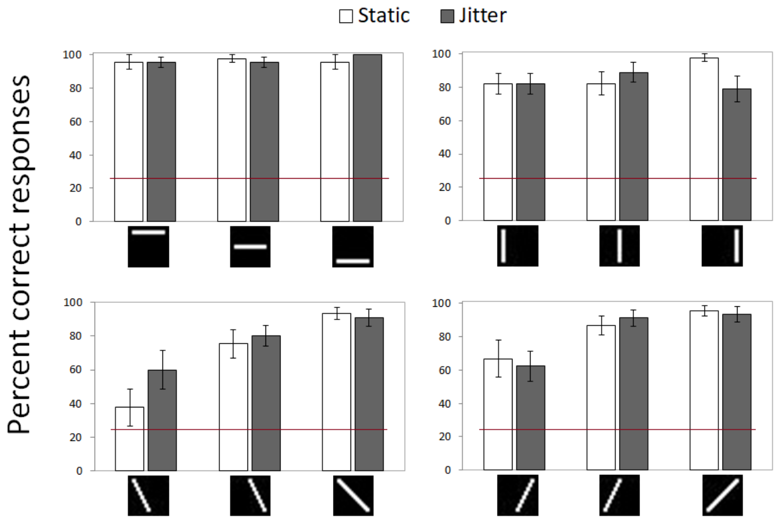

Figure S3. Mean percent correct responses for the orientation of bar recognition in static and jitter conditions. Horizontal red lines indicate the performance level that can be obtained by chance. Participants performed above chance for all conditions. Accuracy was high for the horizontal (top left), vertical (top right) and 45 deg. tilted bars. Performance across 22.5 deg. tilted bars when their top is close to edge was relatively low. There was no difference in performance for static and jittering conditions. effect of position either for horizontal (F(2,16) = 0.15, p = 0.86) or for vertical bars (F(2,16) = 0.6, p = 0.56). However, for the left- and right-tilted bars, position affected the performance (F(2,16) = 15.6, p <0.001; F(2,16) = 7.73, p = 0.004, respectively). Both for the left- and right-tilted bars, the highest performance was observed for the 45 deg. tilted bars and the lowest was for the 22.5 deg. tilted bars whose top is closer to edges, i.e. away from the center of the tongue. Position of the stimulus affected the performance for the left- and right-tilted bars, but not for the horizontal and vertical bars.

Mean RT of correct responses is shown in Figure S4. The data analysis showed that there was no effect of either motion condition (F(1,8) = 0.49 , p = 0.5) or orientation (F(3,24) = 0.39 , p = 0.75) on time required to recognize bars orientation. Also, the interaction between motion and orientation was not significant (F(3,24) = 1.01 , p = 0.4). Because there was no difference between two motion conditons, we averaged the results of static and jitter conditions and compared the effect of position for the further analysis. Results showed that main effect of position for horizontal bar was significant (F(2,16) = 3.98, p = 0.04). However, position did not affect performance for the other orientations (vertical: F(2,16) = 1.1, p = 0.35; left-tilted: F(2,16) = 0.83, p = 0.45; right-tilted: F(2,16) = 0.4, p = 0.67).

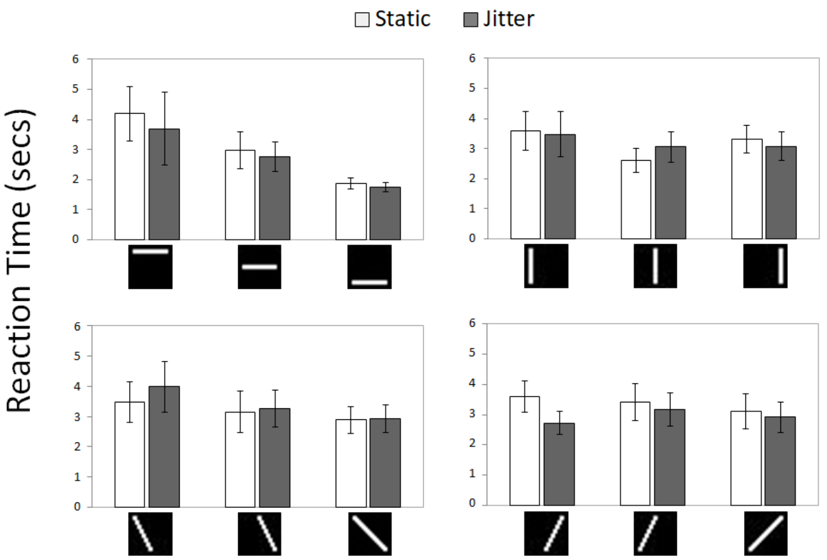

Figure S4. Mean RT of correct responses for the orientation of bar recognition in static and jitter conditions. Ony the main effect of position for horizontal bars was significant which indicates the known effect of asymmetry in sensitivity between the back and tip of the tongue (upper left graph). See text below for a further explanation for this effect.

Summary and Discussion

Results of the jitter study showed that the jittering we apply does not affect recognition of orientation. However, because the task was relatively easy, the performance might have reached to the ceiling for both conditions. For instance, performance for horizontal and 45 deg. tilted bars was already close to 100 percent accuracy (above 90 percent mean accuracy) for both conditions. Therefore, with these results, we cannot eliminate the possibility that jitter affects performance when the stimulus is more complex and more difficult to recognize. To pursue this further, we replicated the study with relatively complex stimuli, letters.

It should also be noted that almost all of our participants reported that they cannot feel any stimulation when a horizontal bar is presented at the back of tongue. Despite this, their performance is close to the maximum because they intuitively and immediately figured out that when they cannot feel any stimulation it should be horizontal bar presented at the back of tongue and responded correctly. The situation also expresses itself in relatively longer RTs in this condition compared to horizontal bars presented on other locations (see Figure S4); participants waited long enough until they were confident that there is no stimulation that they can feel, and reported it as a horizontal line. This observation supports the findings on the low sensitivity of the back of tongue compared to other parts [7,8].

b. Testing the effect of jitter on recognition performance with letter stimuli
Methods
Participants

Six participants (including authors ZP and MUC) took part in the experiment. Three of them participated in the previous jitter study described above. Participants' mean age was 42.2 (*SD* 17.7). No participants had history of any tactile impairment. Two participants were blind. Normally sighted

participants had no history of visual impairments other than the corrected refraction errors. All participants gave written informed consent. Experiments accorded with the Declaration of Helsinki and the protocol and written consent were approved by the Massachusetts Eye and Ear Human Studies Committee.

Apparatus

We used the same apparatus and experimental settings with the previous jitter study with line orientations.

Stimuli

Stimuli were 26 English alphabet capital letters presented in Arial font in 2 different conditions: static and jitter. In the static condition, letters were statically presented on IOD throughout the trial. The first and last two columns of electrodes, and the first two rows and last one row of electrodes were left empty in the static condition, thus the static stimuli only occupied a 16x17 electrode array in order to allocate adequate space in the jitter condition. As in the previous jitter study, the position of each letter in the static condition was considered as the baseline position, and the center of the stimulus was moved to one of the nine electrodes (one on the center and eight neighboring electrodes) in every 50 milliseconds randomly.

Procedure & Data Analysis

The experimental room was identical to the previous study. All participants had prior experience with the BrainPort device and were familiar with the letter or number stimuli presented on IOD of BrainPort Vision Pro, but only in a static condition. All participants were blindfolded and were instructed to put the IOD on the dorsal surface of their tongue, to not move their tongue to use only some parts of it (such as using only tip of the tongue). A stimulus is presented on the IOD to help the participants with adjusting electrical stimulation intensity to a robust but not an uncomfortable level, and their preferences were recorded.

Stimulus presentation and response data collection were identical to the jitter study with line orientation. Each letter was presented in each condition once. There were 52 trials in the experiment (26 letters x 2 conditions (static vs. jitter)). The experimental session lasted approximately 20 minutes. Accuracy and mean RT of correct responses data is analyzed using two-tailed paired-samples Student's t-tests in Jamovi to compare static and jitter conditions.

Results

Individual and mean percent correct responses and RT data are shown in Figure S5 and Figure S6, respectively. Results showed that there was no significant difference in accuracy between static condition (M =43%, SD = 8.7%) and jitter condition (M = 50%, SD = 10.3%; $t(5) = -0.88$, $p = 0.41$). Also, there was no significant difference in RT of the correct responses between static and jitter conditions ($t(5) = 0.51$, $p = 0.63$). Overall, jitter had no effect on the recognition of relatively complex stimuli, letters.

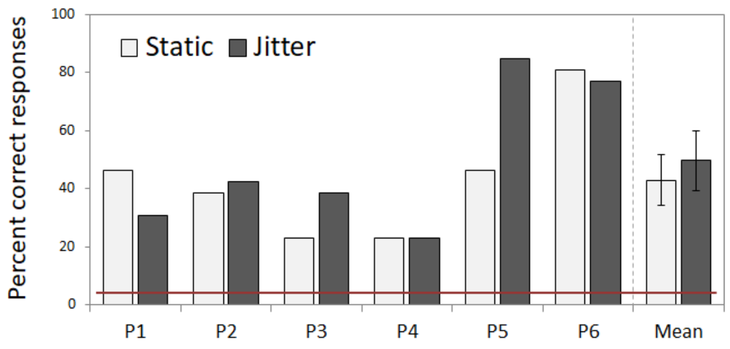

Figure S5. Individual and mean percent correct responses for the letter recognition in static and jitter conditions. Performance is well above chance for both conditions. However, there is no significant difference between static and jitter conditions. The horizontal red line indicates the performance level that can be obtained by chance.

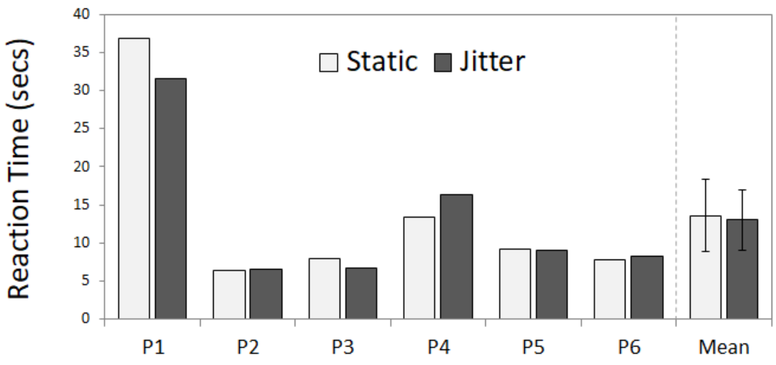

Figure S6. Individual and mean RT of correct responses for the letter recognition in static and jitter conditions. There is no difference between static and jitter conditions.

References

1 Salata, J. A., Raj, J. M. & Doty, R. L. Differential Sensitivity of Tongue Areas and Palate to Electrical Stimulation: A Suprathreshold Cross-Model Matching Study. *Chemical Senses* **16**, 483-489, doi:10.1093/chemse/16.5.483 (1991).

2 Pleasonton, A. K. Sensitivity of the Tongue to Electrical Stimulation. *Journal of Speech, Language, and Hearing Research* **13**, 635-644, doi:10.1044/jshr.1303.635 (1970).

3 Chekhchoukh, A. & Glade, N. Influence of Sparkle and Saccades on Tongue Electro-Stimulation-Based Vision Substitution of 2D Vectors. *Acta Biotheoretica* **60**, 41-53, doi:10.1007/s10441-012-9148-2 (2012).

4 Trulsson, M. & Essick, G. K. Low-threshold mechanoreceptive afferents in the human lingual nerve. *J. Neurophysiol.* **77**, 737-748, doi:10.1152/jn.1997.77.2.737 (1997).

5 Bliss, J. C. & Crane, H. D. Experiments in Tactual Perception. (Stanford Research Institute, Menlo Park, California, Stanford, California, 1965).

6 Chekhchoukh, A., Vuillerme, N., Payan, Y. & Glade, N. in *Engineering in Medicine and Biology Society (EMBS) 2013 35th Annual Internation Conference of IEEE* 3543-3546 (2013).

7 Tyler, M. E., Braun, J. G. & Danilov, Y. P. in *EMBC 2009 Annual International Conference of the IEEE* 559-562 (2009).

8 Moritz, J. J., Turk, P., Williams, J. D. & Stone-Roy, L. M. Perceived Intensity and Discrimination Ability for Lingual Electrotactile Simulation Depends on Location and Orientation of Electrodes. *Front. Neurosci.*, doi:10.3389/fnhum.2017.00186 (2017).
